# Editorial: Immunometabolism in immunological tolerance and regulation: novel mechanisms and clinical interventions

**DOI:** 10.3389/fimmu.2026.1825924

**Published:** 2026-04-01

**Authors:** Yali Zheng

**Affiliations:** 1 Department of Respiratory, Critical Care and Sleep Medicine, Xiang’an Hospital of Xiamen University, School of Medicine, Xiamen University, Xiamen, China; 2 Institute of Chest and Lung Diseases, Xiang’an Hospital of Xiamen University, Xiamen, China

**Keywords:** immune microenvironment, immune tolerance, immunometabolism, metabolic imaging, metabolic reprogramming

The immune system continuously integrates environmental cues, antigenic stimulation, and tissue-specific signals to maintain the balance between immune activation and tolerance. While classical immunology has traditionally focused on receptor signaling pathways and transcriptional regulatory networks, increasing evidence indicates that metabolic circuits represent a fundamental regulatory architecture underlying immune cell function. The emerging field of immunometabolism therefore investigates how metabolic pathways intersect with immune signaling to shape immune responses. In this context, immune tolerance, which is once considered a passive consequence of immune suppression, is now recognized as an actively maintained state constrained by metabolic programs.

Here in this Research Topic, we propose a conceptual framework in which immune tolerance is governed by interconnected immunometabolic checkpoints operating at multiple biological levels. These checkpoints encompass intrinsic metabolic programs within immune cells, metabolic conditions within tissue microenvironments, and systemic metabolic regulation together with emerging technological advances. Together, these interconnected immunometabolic checkpoints form a regulatory architecture that governs immune homeostasis across cellular, tissue, and systemic contexts.

## Intrinsic metabolic programs determine immune cell fate

At the most cellular level, immune tolerance is shaped by metabolic programs operating within immune cells. Distinct immune cell states are closely associated with characteristic metabolic configurations, including effector activation, functional exhaustion, and regulatory phenotypes. Within the adaptive immune system, T cell subsets exhibit specialized and functionally requisite metabolic configurations. Regulatory T cells (Tregs) rely on metabolic programs centered on mitochondrial oxidative phosphorylation and fatty acid oxidation to maintain stability and suppressive capacity, illustrating how metabolic flexibility underpins sustained immune tolerance (Lu et al.). Conversely, in pathological states such as severe COVID-19, aberrant metabolic reprogramming in effector T cells is directly linked to dysfunction (Long et al.). The expansion of activated CD38+HLA-DR+ T cell subsets is associated with NAD+ metabolism dysregulation, which correlates with their exhausted phenotype and poor clinical prognosis, highlighting how metabolic disruption can precipitate a loss of functional competence.

This principle extends to innate immunity. For instance, the polarization of macrophages into pro-inflammatory (M1) or anti-inflammatory/repair (M2) phenotypes is governed by distinct metabolic shifts, with mitochondrial integrity and related genes being critical for directing this functional switch, as evidenced in chronic inflammatory diseases (Zhang et al.).

Collectively, these findings establish that intrinsic immunometabolic checkpoints are pivotal in determining whether an immune response progresses towards inflammatory escalation, stabilizes into a regulatory tolerant state, or devolves into dysfunctional exhaustion.

## Tissue metabolic environments shape immune tolerance

Beyond cell-intrinsic metabolism, metabolic conditions within tissue microenvironments exert profound influences on immune tolerance. Nutrient availability, oxygen tension, and inflammatory metabolites collectively shape immune cell behavior and functional outcomes. In complex immune contexts such as organ transplantation, the metabolic landscape of the graft microenvironment can profoundly influence immune responses (Lan et al.). Interactions between immune cells, cytokine networks, and metabolic perturbations contribute to the establishment of either graft rejection or immune tolerance. Such observations highlight how metabolic cues within tissues can modulate immune regulatory pathways.

Spatial organization within tissues further contributes to immune regulation. Adhesion molecules such as integrin αL, a component of the LFA-1 complex, regulate leukocyte migration, immune synapse formation, and intercellular communication (Wang et al.). By coordinating immune cell trafficking and interactions within specific metabolic niches, these molecules help organize immune responses within tissue microenvironments.

Thus, immune tolerance arises not solely from intracellular metabolic programs but from the dynamic integration of immune signaling with the metabolic architecture of tissues.

## Systemic metabolism and technological advances

At a broader level, host metabolic status and technological innovations are expanding our ability to understand and manipulate immune tolerance. Recent advances in molecular imaging technologies now allow researchers to visualize immune cell metabolism *in vivo* (Li et al.). Techniques such as optical imaging, nuclear medicine imaging, and magnetic resonance imaging can monitor immune cell migration, metabolic activity, and therapeutic responses in real time. A review included in this Research Topic highlights the application of molecular imaging in studying immune tolerance and immune metabolism, emphasizing how these approaches enable dynamic visualization of immune processes within living organisms.

Importantly, metabolic imaging can detect functional alterations in immune cells before structural changes become apparent, thereby providing new opportunities for early disease diagnosis and treatment monitoring. As metabolic tracers and multimodal imaging approaches continue to develop, immunometabolism is increasingly evolving from a mechanistic research field into a translational discipline capable of informing clinical decision-making.

## Therapeutic implications and future directions in immunometabolic regulation

Understanding immunometabolic checkpoints opens new avenues for therapeutic intervention in immune-mediated diseases. Multiple metabolic pathways may serve as potential targets for modulating immune responses. Manipulating key metabolic regulators such as mTOR signaling, mitochondrial metabolism, or lipid utilization may reshape immune cell differentiation and restore immune balance (Zhang et al.). In autoimmune diseases, metabolic interventions may enhance immune tolerance, whereas in cancer, targeting the metabolic landscape of the tumor microenvironment may help restore effective anti-tumor immunity (Habiballah et al.). Furthermore, the integration of metabolic imaging with biomarker discovery may facilitate personalized therapeutic strategies based on individual immunometabolic profiles.

Despite substantial progress, many aspects of metabolic regulation of immune tolerance remain incompletely understood. Future research should aim to integrate multi-omics approaches—including metabolomics, transcriptomics, spatial biology, and advanced imaging technologies—to construct a comprehensive immunometabolic map of immune regulation. Such efforts will help identify key metabolic checkpoints that determine immune cell fate and clarify how systemic metabolic states such as aging, obesity, or infection reshape immune tolerance. Ultimately, the integration of metabolic biology with immunology may redefine our understanding of immune regulation and provide new strategies for restoring immune homeostasis in human disease.

